# Health inequalities in very old age: Continuity, accumulation or convergence?

**DOI:** 10.1093/eurpub/ckac129.032

**Published:** 2022-10-25

**Authors:** J Wenner, J Fey, S Zank, M Wagner

**Affiliations:** University of Cologne, Köln, Germany

## Abstract

**Background:**

Health inequalities are well documented empirically. However, it is unclear whether health inequalities persist in very old age (continuity), whether they accumulate steadily (accumulation), or whether they even attenuate in old age (convergence) - not least because of social inequalities in life expectancy, which make it less likely for individuals with lower social status to reach old age at all. The aim of this study is to empirically test these three hypotheses.

**Methods:**

The analyses are based on representative cross-sectional data from 1,863 very old people and panel data from 912 participants in the 1st and 2nd wave (W1, W2) living in North Rhine-Westphalia the largest federal state in Germany (NRW80+ study). Health outcomes of the analyses are subjective health, multimorbidity and need for long-term care. Indicators of socioeconomic status (SES) are education, occupational status, and net equivalent income. Regression models (linear, logistic, ordinal) are used to analyze cross-sectional and longitudinal data. Panel selectivity is also considered by means of a failure model.

**Results:**

Cross-sectional findings show health inequalities for all SES variables: persons with low education and low income have poorer subjective health and higher need for care. Low status is associated with higher need for care. Preliminary results from longitudinal analyses show a slight increase in health inequality: low income and low status are associated with higher multimorbidity and low education with higher dependency on long-term care at W2.

**Conclusions:**

Despite the socially conditioned unequal chances of reaching old age, health inequality is still present in very old age and even increases slightly over time. The results argue against the convergence hypothesis and in favor of the continuity or even the accumulation hypothesis. A better understanding of the mechanisms leading to the persistent inequality is needed to development targeted interventions also in old age.

**Key messages:**

• Health inequalities persist and even increase in very old age (80+).

• It is imperative that the oldest old – an increasingly large population group – be considered when designing strategies to reduce health inequalities.

